# Ocular Manifestations of Biopsy-Proven Pulmonary Sarcoidosis in Korea

**DOI:** 10.1155/2018/9308414

**Published:** 2018-02-11

**Authors:** Seung Yong Choi, Jae Hoon Lee, Jae-Yon Won, Jeong Ah Shin, Young-Hoon Park

**Affiliations:** ^1^Department of Ophthalmology and Visual Science, College of Medicine, The Catholic University of Korea, Seoul, Republic of Korea; ^2^Catholic Institute for Visual Science, College of Medicine, The Catholic University of Korea, Seoul, Republic of Korea

## Abstract

**Purpose:**

To investigate the clinical features and ocular manifestations of biopsy-proven pulmonary sarcoidosis in Korea.

**Methods:**

55 patients diagnosed with pulmonary sarcoidosis by bronchoscopic or excisional biopsy were included. By retrospective clinical chart review, we investigated features of uveitis, ocular and systemic treatments, visual acuity, angiotensin-converting enzyme level, chest radiography, and pulmonary function tests. Clinical features were analyzed by presence of uveitis, site of biopsy, and first manifested sign of sarcoidosis.

**Results:**

The group with uveitis (*n* = 39) presented with higher systemic (71.8%) and immunosuppressive treatment rates (35.9%) than the group without uveitis (31.3%, 0%, resp.) (*P* = 0.007, *P* = 0.005, resp.). There were no significant differences in clinical features, including systemic treatment rate, by type of biopsy. Of 39 patients with uveitis, the group with ocular manifestation as a first sign of sarcoidosis showed higher systemic and immunosuppressive treatment rates (88.9%, 55.6%) compared to the group with pulmonary manifestation as a first sign (57.1%, 19.0%) (*P* = 0.037, *P* = 0.018, resp.).

**Conclusions:**

In patients with biopsy-proven pulmonary sarcoidosis, the presence of ocular involvement and uveitis as a first sign could be significant factors associated with higher systemic treatment rate, especially with immunosuppressive agents. Biopsy site determined by location and size had no influence on clinical features.

## 1. Introduction

Sarcoidosis is an idiopathic chronic inflammatory disease that affects multiple organs [1]. Approximately 30% to 60% of patients with sarcoidosis develop ophthalmic changes, and granulomatous uveitis is a common manifestation of ocular sarcoidosis [2–4]. Ocular sarcoidosis can occur without apparent systemic involvement, and ocular involvement can be the main site of disease without any significant systemic involvement [5].

It is difficult to confirm the diagnosis in patients suspected to have ocular sarcoidosis without biopsy findings or significant extraocular manifestations, such as bilateral hilar lymphadenopathy, increased serum angiotensin-converting enzyme (ACE) level, and other laboratory findings.

Sarcoidosis can be convincingly diagnosed by typical histological findings of noncaseating granuloma in biopsy specimens and the exclusion of previous exposure to specific pathogens that cause granulomatous inflammation, such as acid-fast bacilli [4]. However, a biopsy of intraocular tissues is not commonly performed because of the high risk of complications. A biopsy of other tissues, such as lung, skin, and peripheral lymph nodes, is usually performed for diagnosis [6]. Bronchoscopy-assisted biopsy techniques, such as endobronchial ultrasound-guided transbronchial needle aspiration (EBUS-TBNA), are often used to diagnose sarcoidosis [7, 8].

In previous studies, the ocular involvement of biopsy-proven sarcoidosis has been reported in 21–55% of cases [9–11]. Biopsy sources include the lung, skin, lymph nodes, and other organs. In another study, the ocular involvement of confirmed pulmonary sarcoidosis was reported in 74% of cases [9]. Differences in the ocular involvement rate can be explained by regional variation in the prevalence of sarcoidosis, HLA typing, clinical practice, and other factors [11, 12].

In this study, we investigated the clinical features and ocular manifestations of pulmonary sarcoidosis confirmed by endobronchial ultrasound-guided transbronchial needle aspiration (EBUS-TBNA) or excisional biopsy of hilar or extrapulmonary lesion in Korean patients and compared the findings with other reported features of biopsy-proven sarcoidosis.

## 2. Methods

Patients who were diagnosed with pulmonary sarcoidosis based on biopsy findings (EBUS-TBNA, excisional biopsy of hilar lesion, or excisional biopsy of extrapulmonary lesion) between January 2011 and December 2015 were included in this retrospective single-center study. Patients with evidence of other granulomatous disease or other causes of uveitis (such as positive tuberculin test or positive syphilis serology) and patients with sarcoidosis in other major organs (e.g., the brain, heart) were excluded. Sarcoid nodules in the skin or other peripheral lymph nodes (e.g., the axilla, neck) were not considered exclusion criteria. Histological findings of all patients showed noncaseating granuloma without acid-fast bacilli and significant lymphocyte cell populations with multinucleated cells [7, 13].

Biopsy type was determined by pulmonary specialists based on the site of the most easily accessible lesion. Most patients with hilar lesions and without extrapulmonary lesions by bronchoscopy underwent EBUS-TBNA. However, patients without any accessible hilar lesion by bronchoscopy underwent excisional biopsy of hilar lesion by thoracoscopy. If a patient had extrapulmonary lesions that were more easily accessible by excisional biopsy than bronchoscopic biopsy, an excisional biopsy of the extrapulmonary lesion was performed for histological confirmation.

All patients reported to the ophthalmology department and underwent comprehensive ophthalmologic examinations, including best-corrected visual acuity (of the manifested eye or of the more severely affected eye), anterior segment slit-lamp examinations, intraocular pressure, gonioscopy, funduscopy with wide-field contact lens, optical coherence tomography (OCT), and fluorescein angiography (FAG). Sarcoid uveitis was diagnosed by characteristic ocular signs of sarcoidosis based on the International Workshop on Ocular Sarcoidosis (IWOS) guidelines of 2009 [14]: (1) mutton-fat keratic precipitates and iris nodules; (2) nodules in the trabecular meshwork and tent-shaped peripheral anterior synechia; (3) snowball or string-of-pearls vitreous opacities; (4) nodular periphlebitis; (5) multiple chorioretinal lesions; (6) optic disc nodule(s)/granuloma(s) and/or solitary choroidal nodule; and (7) bilaterality.

We classified the type of uveitis into anterior, intermediate, and posterior uveitis. Anterior uveitis was defined as signs of inflammation in anterior chamber without vitreal or retinal change. Intermediate uveitis was defined as vitreal cell or vitreal opacity without retinal change. Posterior uveitis was defined as uveitis with retinal change found by either funduscopy or angiography. The patients with angiographic change including focal retinal phlebitis, disc leakage, or diffuse vascular leak were classified as having posterior uveitis. The patients with only vague vascular leak at far periphery, focal arterial or capillary leak, or angiographic CME were not classified as having posterior uveitis.

By retrospective clinical chart review, demographic data, number of uveitis attacks, type of uveitis, Snellen visual acuity of worse eye at last follow-up, complications, and treatments were investigated. Extraocular manifestations, results of serum angiotensin-converting enzyme (ACE) assay at time of diagnosis, chest radiography including plain X-ray or computed tomography (CT) at time of diagnosis, and pulmonary function tests (PFT) at last follow-up were also assessed.

Statistical analysis was carried out with SPSS 19.0 software (SPSS Inc., Chicago, IL, USA), and a *P* value < 0.05 was defined as statistically significant.

## 3. Results

A total of 55 patients with biopsy-proven pulmonary sarcoidosis were included and had a mean age of 53.6 ± 14.0 and female dominance (45 over 55). The group of biopsy-proven pulmonary sarcoidosis included 39 patients with ocular manifestations (70.9%), 49 patients with bilateral hilar lymphadenopathy (BHL) (89.1%), 37 patients with increased ACE level (67.3%), and 1 patient with abnormal pulmonary function test who showed no ocular manifestations. Of 33 patients with systemic treatments, 19 (34.5%) were treated by systemic steroids and 14 (25.5%) were treated by immunosuppressive agents. A total of 22 patients (40.0%) did not require systemic treatment.

The 39 patients with ocular involvement had a mean age of 57.1 ± 15.5 years and were predominantly female (35 out of 39). There was a statistically significant difference in mean age (48.0 ± 13.7 years) and sex ratio (10 out of 16) of patients without and with ocular involvement (*P* = 0.045, *P* = 0.048, resp.). In the group with ocular involvement, 87.2% had BHL by chest radiography (34 patients), 74.4% had increased ACE (29 patients), mean ACE level was 77.1 ± 40.9, and mean visual acuity (Snellen) was 0.83 ± 0.19. In the group with no ocular involvement, 15 patients had BHL (93.8%) and 8 patients had increased ACE (50.0%); mean ACE level was 61.7 ± 33.9 and mean visual acuity (Snellen) was 0.90 ± 0.14. These results showed no statistically significant difference between the 2 groups (*P* = 0.66, *P* = 0.12, *P* = 0.19, and *P* = 0.15, resp.). There was a statistically significant difference in the rate of systemic treatment (28 patients, 71.8% of the group with ocular involvement and 5 patients, 31.3% of the group with no ocular involvement) (*P* = 0.007). The rate of immunomodulative treatment was also significantly different between the 2 groups (14 patients, 35.9% of the group with ocular involvement and no patients in the group with no ocular involvement) (*P* = 0.005) (Table 1).

There were 3 patients who underwent intravitreal or periocular injection of steroid for macular edema (1 patient with unilateral macular edema and 2 patients with bilateral macular edema). One of the patients with macular edema underwent pars plana vitrectomy for diagnosed refractory unilateral macular edema. There were no patients with visually significant epiretinal membrane which showed over 300 *μ*m of central macular thickness. All the complicated patients were diagnosed as having sarcoidosis by EBUS-TBNA.

38 patients with biopsy-confirmed sarcoidosis by EBUS-TBNA (group A), 8 with excisional biopsy of a hilar lesion (group B), and 9 with biopsy of extrapulmonary lesions (group C) were investigated for comparison. In group C, biopsy of extrapulmonary lesions were done for the skin (4 patients) and axillary lymph node (5 patients). The three groups (A, B, and C) did not show differences in mean age (53.8 ± 13.9, 53.5 ± 15.5, and 57.9 ± 16.2, resp.) or female sex ratio (31 of 38, 5 of 8, and 9 of 9, resp.) (*P* = 0.96 and *P* = 0.14, resp.). The measurements of groups A, B, and C did not show significant differences in positive rate of BHL by radiography (92.1%, 87.5%, and 77.8%, resp.), rate of patients with increased ACE (68.4%, 62.5%, and 66.7%, resp.), mean ACE level (73.8 ± 40.0, 65.9 ± 36.4, and 72.2 ± 43.8, resp.), and Snellen visual acuity at last follow-up (0.82 ± 0.23, 0.92 ± 0.13, and 0.83 ± 0.25, resp.) (all *P* values > 0.10). Although there was no statistical significance, the rates of ocular involvement, systemic treatment, and immunomodulative treatment were higher in group C (100%, 88.9%, and 66.7%, resp.) than those in groups A (65.8%, 55.3%, and 23.7%, resp.) and B (62.5%, 50.0%, and 12.5%, resp.) (*P* = 0.11, *P* = 0.15, and *P* = 0.29, resp.) (Table 2).

In the group with ocular involvement (*n* = 39), 18 patients presented with ocular symptoms as an initial manifestation of sarcoidosis and visited the department of ophthalmology first. Based on site of uveitis, 12 patients (30.8%) manifested only anterior uveitis, 10 patients (25.6%) manifested intermediate uveitis without posterior involvement, and 17 patients (43.6%) manifested posterior or panuveitis. The rate of bilateral involvement of uveitis was 74.4% (29 patients). Regarding uveitis complications, 7 patients had cystoid macular edema, and 2 patients had uveitic glaucoma. Six patients underwent intravitreal injection treatment, and 1 patient underwent pars plana vitrectomy for control of uveitis or treatment of complications.

Of the 18 patients with ocular involvement as an initial manifestation of sarcoidosis (ocular manifestation first group), the mean age was 57.4 ± 15.3, and females were predominant (17 over 18). There was no significant difference in the mean age (51.1 ± 12.0) or female sex ratio (18 over 21) compared with the ocular manifestation first group in the 21 patients with extraocular involvement (extraocular manifestation first group) as the initial manifestation of sarcoidosis (*P* = 0.45 and *P* = 0.61, resp.). The ocular manifestation first group showed no significant difference in rate of positive bilateral hilar lymphadenopathy (83.3%), rate of increased ACE level (72.2%), mean ACE level (82.4 ± 46.5), rate of ocular complications (22.2%), or final visual acuity (0.83 ± 0.19) compared with the extraocular manifestation first group (90.5%, 76.2%, 72.6 ± 36.0, 23.8%, and 0.90 ± 0.14, resp.) (all *P* values > 0.05). The distribution of characteristic ocular signs of sarcoidosis based on IWOS guidelines [14] did not show significant differences between the 2 groups (all *P* values > 0.05). In the ocular manifestation first group, there were significantly more frequent prescriptions of systemic steroids (88.9%) or immunomodulative agents (55.6%) than in the extraocular manifestation first group (57.1% and 19.0%, resp.) (*P* = 0.037 and *P* = 0.018, resp.) (Table 3).

## 4. Discussion

This study indicates that the presence of uveitis and uveitis as a first manifestation of biopsy-proven pulmonary sarcoidosis could be significant factors associated with higher systemic treatment rate, especially with immunomodulative agents. The biopsy site was determined by accessibility, and presence of extrapulmonary lesion did not have an effect on clinical features or systemic treatment rate.

Of 55 patients with biopsy-confirmed pulmonary sarcoidosis, most did not have any respiratory signs or symptoms that limited daily life. Some patients experienced mild cough, and 1 patient without uveitis showed decreased pulmonary function, but the daily activity of this patient was not severely limited. Although patients with uveitis showed some complications and treatment history, their mean visual acuity at last follow-up was up to 20/25 by Snellen chart. These findings are similar to those of previous reports about the visual prognosis of sarcoid uveitis [15–17].

Despite similar respiratory and visual prognoses, patients with ocular involvement had significantly higher treatment rates with systemic steroids or immunomodulative agents in this study. This might have been affected by clinical settings or other factors, but the results suggest that ocular involvement could be of clinical interest for systemic treatment. In a previous study [17], there was a higher systemic treatment rate in patients with ocular and systemic sarcoidosis compared to patients with isolated ocular sarcoidosis. The disagreement between the two studies could be due to the locations of systemic involvement by sarcoidosis. In this study, most extrapulmonary manifestations were limited to the skin and peripheral lymph nodes, except in 1 patient with development of cardiac sarcoidosis. A previous study [17] included a number of patients with major organ involvement (the liver, cardiac, CNS, and kidney), and differences in disease severity could result in disagreements in treatment rate.

Bronchoscopy-assisted biopsy is reported to have about 70% diagnostic yield for pathological confirmation of sarcoidosis [18]. As a new diagnostic tool, endobronchial ultrasound-guided transbronchial needle aspiration (EBUS-TBNA) showed 74.5–86% [19, 20] diagnostic yield and is considered to have a higher diagnostic value than transbronchial lung biopsy (TBLB). High diagnostic yield, easier approach to lesions, and outpatient-based procedure were reasons for the higher distribution of patients in the EBUS-TBNA group.

Higher serum ACE could indicate higher monocytic cell line activity or granulomatous inflammation [21]. The predictive value of ACE for diagnosing sarcoidosis is 47%, lower than that of chest radiographic findings [22, 23]. Although there was no statistical significance, the rate of increased ACE level and mean ACE level was slightly higher in the group with ocular involvement in this study. It is possible that, in patients with sarcoidosis, higher serum ACE level could indicate higher disease activity and increased vulnerability for ocular manifestations. Further studies are needed to investigate the clinical significance of ACE level in sarcoidosis with and without ocular involvement.

Comparison of groups with different biopsy types used for diagnosis showed that extraocular and extrapulmonary manifestations did not influence ocular manifestation, ACE level, or systemic treatment rate. Although there were few patients, this study suggests that, except for major organs, extraocular manifestations are insufficient to indicate the disease activity of sarcoidosis.

Kim et al. [24] reported that 7.1% of Korean patients with noninfectious uveitis were diagnosed with sarcoidosis. There was ocular involvement by sarcoidosis in 26.5% of cases, and uveitis was an initial manifestation in 13.7% of cases. The female ratio was 80.7%, and bilateral involvement by uveitis occurred in 87.1% of cases. Lee et al. [10] also reported a dominant female prevalence (86%) and high bilateral involvement (77%).

In this study, female prevalence (81.6%) and bilateral involvement rate (74.4%) were similar, but the ocular involvement rate and uveitis as initial manifestation were much higher than in previous studies [10] (65.8% and 46.2%, resp.). Routine peripheral fundus examination with contact lenses and wide-field fluorescein angiography could explain the higher incidence of uveitis, especially posterior uveitis or panuveitis. These exams can identify peripheral retinal granulomatous lesions and mild disc or vascular leakage, which cannot be identified by routine fundus exam. Further study will be needed for the sensitivity of wide-field imaging in the diagnosis of sarcoid uveitis.

The ocular involvement rate of transbronchial biopsy-proven sarcoidosis was 65.8% in this study. This is comparable with the rate reported in previous studies (21–74%) [9–12]. Differences in ocular involvement rate are explained by regional variations in the prevalence of sarcoidosis, HLA typing, clinical practice, and other factors [11, 12]. Since previous studies [9, 11] included the patients with sarcoidosis involving major organs other than the lung, the inclusion of pulmonary sarcoidosis without other major organ involvement in this study could be a reason of difference in the ocular involvement rate. Dominant type of uveitis and bilateral involvement are variables between studies of confirmed sarcoidosis [9–12, 25, 26] (Table 4).

In this study, we only included the patients with biopsy-proven pulmonary sarcoidosis without other major organ involvement. A careful interpretation for this study would be needed because of the limited disease severity and number of included subjects. Laboratory markers, like lysozyme and soluble interleukin 2, were not routine examinations for the patients with sarcoidosis in our clinical setting. The lack of results of laboratory markers, which could indicate the activity or burden of sarcoidosis [22], could be a limitation of this study. For some subjects, the follow-up period was relatively short in this study. Further study will be needed for the long-term incidence of uveitis in the biopsy-confirmed pulmonary sarcoidosis. In conclusion, Korean patients with biopsy-proven pulmonary sarcoidosis showed a high ocular involvement rate of 65.8% with dominant female prevalence (78.8%). Uveitis showed a high bilateral involvement rate (74.4%) and a relatively high ocular manifestation rate as initial manifestations (46.2%) in this study. The presence of ocular manifestations and uveitis as a first sign of disease could be significant factors associated with higher treatment rate. The accessibility of pulmonary lesions and extraocular manifestations of minor organs are not proper markers of disease activity or decisive factors for treatment.

## Figures and Tables

**Table 1:** Clinical features of patients with biopsy-proven pulmonary sarcoidosis according to ocular involvement.

	Ocular involvement (*n* = 39)	No ocular involvement (*n* = 16)	*P* value
Mean age	57.1 ± 15.5	48.0 ± 13.7	0.045
Female/male ratio	35/4	10/6	0.048
BHL^a^ in radiography	34 (87.2%)	15 (93.8%)	0.66
Increased ACE^b^	29 (74.4%)	8 (50.0%)	0.12
ACE^b^ level	77.1 ± 40.9	61.7 ± 33.9	0.19
Visual acuity at last visit	0.83 ± 0.19	0.90 ± 0.14	0.15
Systemic treatment	28 (71.8%)	5 (31.3%)	0.007
Immunosuppression	14 (35.9%)	0 (0.0%)	0.005

^a^Bilateral hilar lymphadenopathy; ^b^angiotensin-converting enzyme.

**Table 2:** Clinical features by type of biopsy for confirmation of sarcoidosis.

	Transbronchial (*n* = 38)	Excisional (lung) (*n* = 8)	Extrapulmonary (*n* = 9)	*P* value
Mean age	53.8 ± 13.9	53.5 ± 15.5	57.9 ± 16.2	0.96
Female/male ratio	31/7	5/3	9/0	0.14
Ocular involvement	25 (65.8%)	5 (62.5%)	9 (100%)	0.11
BHL^a^ in radiography	35 (92.1%)	7 (87.5%)	7 (77.8%)	0.46
Increased ACE^b^	27 (68.4%)	5 (62.5%)	6 (66.7%)	0.95
ACE^b^ level	73.8 ± 40.0	65.9 ± 36.4	72.2 ± 43.8	0.87
Visual acuity at last visit	0.82 ± 0.23	0.92 ± 0.13	0.83 ± 0.25	0.75
Systemic treatment	21 (55.3%)	4 (50.0%)	8 (88.9%)	0.15
Immunosuppression	9 (23.7%)	1 (12.5%)	6 (66.7%)	0.29

^a^Bilateral hilar lymphadenopathy; ^b^angiotensin-converting enzyme.

**Table 3:** Comparison of uveitis patients with biopsy-confirmed pulmonary sarcoidosis by initial manifestation.

	Ocular manifestation first group (*n* = 18)	Extraocular manifestation first group (*n* = 21)	*P* value
Mean age	57.4 ± 15.3	54.1 ± 12.0	0.45
Female/male ratio	17/1 (94.4%)	18/3 (85.7%)	0.61
BHL^a^ in radiography	15 (83.3%)	19 (90.5%)	0.65
Increased ACE^b^	13 (72.2%)	16 (76.2%)	0.78
ACE^b^ level	82.4 ± 46.5	72.6 ± 36.0	0.47
Ocular complications	4 (22.2%)	5 (23.8%)	0.61
Visual acuity at last visit	0.83 ± 0.19	0.90 ± 0.14	0.15
Ocular involvement			
Mutton-fat KP^c^	16 (88.9%)	14 (66.7%)	0.10
Angle change	2 (11.1%)	3 (14.3%)	0.58
Vitreous opacity	5 (27.8%)	5 (23.8%)	0.53
Nodular phlebitis	13 (72.2%)	13 (61.9%)	0.37
Multiple choroidal lesions	7 (38.9%)	6 (28.6%)	0.36
Optic disc lesion	3 (16.7%)	5 (23.8%)	0.44
Bilaterality	16 (88.9%)	13 (61.9%)	0.07
Systemic treatment	16 (88.9%)	12 (57.1%)	0.037
Immunosuppression	10 (55.6%)	4 (19.0%)	0.018

^a^Bilateral hilar lymphadenopathy; ^b^angiotensin-converting enzyme; ^c^keratic precipitate.

**Table 4:** Comparison of method of diagnosis confirmation and prevalence and type of uveitis in studies of confirmed sarcoidosis.

	This study	Evans et al. [9]	Lee et al. [10]	Sungur et al. [11]	Fakin et al. [12]	Febvay et al. [24]	Birnbaum et al. [25]
Method of diagnosis (biopsy site)	Biopsy (variable)	Biopsy (variable)	Biopsy (variable)	Biopsy (unknown)	ATS/ERS^a^ criteria	Biopsy (variable)	Biopsy (variable)
Prevalence of uveitis (number of subjects)	71% (55)	41% (81)	21% (104)	55% (47)	100%^b^ (53)	100%^b^ (83)	100%^b^ (63)
Bilaterality	74.4%	Unknown	77%	100%	79%	75%	89%
Type of uveitis							
Anterior	30.8%	52%	41%	15.4%	21%	19%	30%
Intermediate	25.6%	12%	31%	46.2%	38%	18%	11%
Posterior/panuveitis	43.6%	39%	28%	38.5%	41%	63%	59%

^a^ATS/ERS criteria: American Thoracic Society/European Respiratory Society criteria; ^b^only uveitis patients were included.

## References

[1] Newman L. S., Rose C. S., Maier L. A. (1997). Sarcoidosis. *The New England Journal of Medicine*.

[2] Jabs D. A., Johns C. J. (1986). Ocular involvement in chronic sarcoidosis. *American Journal of Ophthalmology*.

[3] Kawaguchi T., Hanada A., Horie S., Sugamoto Y., Sugita S., Mochizuki M. (2007). Evaluation of characteristic ocular signs and systemic investigations in ocular sarcoidosis patients. *Japanese Journal of Ophthalmology*.

[4] Stavrou P., Linton S., Young D. W., Murray P. I. (1997). Clinical diagnosis of ocular sarcoidosis. *Eye*.

[5] Ohara K., Okubo A., Sasaki H., Kamata K. (1992). Intraocular manifestations of systemic sarcoidosis. *Japanese Journal of Ophthalmology*.

[6] Koerner S. K., Sakowitz A. J., Appelman R. I., Becker N. H., Schoenbaum S. W. (1975). Transbronchial lung biopsy for the diagnosis of sarcoidosis. *The New England Journal of Medicine*.

[7] Davis G. S. (2014). The role of endobronchial ultrasound-guided transbronchial needle aspiration (EBUS TBNA) in the diagnosis of sarcoidosis. *Cancer Cytopathology*.

[8] Ohara K., Okubo A., Kamata K., Sasaki H., Kobayashi J., Kitamura S. (1993). Transbronchial lung biopsy in the diagnosis of suspected ocular sarcoidosis. *Archives of Ophthalmology*.

[9] Evans M., Sharma O., LaBree L., Smith R. E., Rao N. A. (2007). Differences in clinical findings between Caucasians and African Americans with biopsy-proven sarcoidosis. *Ophthalmology*.

[10] Lee S. Y., Lee H. G., Kim D. S., Kim J. G., Chung H., Yoon Y. H. (2009). Ocular sarcoidosis in a Korean population. *Journal of Korean Medical Science*.

[11] Sungur G., Hazirolan D., Bilgin G. (2013). Pattern of ocular findings in patients with biopsy-proven sarcoidosis in Turkey. *Ocular Immunology and Inflammation*.

[12] Fakin A., Terčelj M., Vidović Valentinčič N. (2016). Frequency of IWOS suggestive ocular signs in Slovenian uveitis patients with confirmed pulmonary sarcoidosis. *Ocular Immunology and Inflammation*.

[13] Zaman S. S., Elshami A., Gupta P. K. (1995). Bronchoalveolar lavage cytology in pulmonary sarcoidosis. *Acta Cytologica*.

[14] Herbort C. P., Rao N. A., Mochizuki M., the members of the Scientific Committee of the First International Workshop on Ocular Sarcoidosis (IWOS) (2009). International criteria for the diagnosis of ocular sarcoidosis: results of the first International Workshop on Ocular Sarcoidosis (IWOS). *Ocular Immunology and Inflammation*.

[15] Edelsten C., Pearson A., Joynes E., Stanford M. R., Graham E. M. (1999). The ocular and systemic prognosis of patients presenting with sarcoid uveitis. *Eye*.

[16] Lobo A., Barton K., Minassian D., du Bois R. M., Lightman S. (2003). Visual loss in sarcoid-related uveitis. *Clinical and Experimental Ophthalmology*.

[17] Rochepeau C., Jamilloux Y., Kerever S. (2016). Long-term visual and systemic prognoses of 83 cases of biopsy-proven sarcoid uveitis. *British Journal of Ophthalmology*.

[18] Navani N., Booth H. L., Kocjan G. (2011). Combination of endobronchial ultrasound-guided transbronchial needle aspiration with standard bronchoscopic techniques for the diagnosis of stage I and stage II pulmonary sarcoidosis. *Respirology*.

[19] Dziedzic D. A., Peryt A., Orlowski T. (2017). The role of EBUS-TBNA and standard bronchoscopic modalities in the diagnosis of sarcoidosis. *The Clinical Respiratory Journal*.

[20] Gupta D., Dadhwal D. S., Agarwal R., Gupta N., Bal A., Aggarwal A. N. (2014). Endobronchial ultrasound-guided transbronchial needle aspiration vs conventional transbronchial needle aspiration in the diagnosis of sarcoidosis. *Chest*.

[21] Bénéteau-Burnat B., Baudin B. (1991). Angiotensin-converting enzyme: clinical applications and laboratory investigations on serum and other biological fluids. *Critical Reviews in Clinical Laboratory Sciences*.

[22] Baarsma G. S., La Hey E., Glasius E., de Vries J., Kijlstra A. (1987). The predictive value of serum angiotensin converting enzyme and lysozyme levels in the diagnosis of ocular sarcoidosis. *American Journal of Ophthalmology*.

[23] Lynch J. P. (2003). Computed tomographic scanning in sarcoidosis. *Seminars in Respiratory and Critical Care Medicine*.

[24] Kim T. W., Chung H., Yu H. G. (2008). Clinical features in Korean patients with sarcoid uveitis. *Journal of the Korean Ophthalmological Society*.

[25] Birnbaum A. D., Oh F. S., Chakrabarti A., Tessler H. H., Goldstein D. A. (2011). Clinical features and diagnostic evaluation of biopsy-proven ocular sarcoidosis. *Archives of Ophthalmology*.

[26] Febvay C., Kodjikian L., Maucort-Boulch D. (2015). Clinical features and diagnostic evaluation of 83 biopsy-proven sarcoid uveitis cases. *British Journal of Ophthalmology*.

